# Timely Surgical Approaches for Pediatric Epilepsy Resistant to Medication

**DOI:** 10.26502/jsr.10020513

**Published:** 2026-08-04

**Authors:** Tyler Williams, Spencer Collins, Edgar Sanchez, Devendra K. Agrawal

**Affiliations:** Department of Translational Research, College of Osteopathic Medicine of the Pacific, Western University of Health Sciences, Pomona, California 91766 USA

**Keywords:** Artificial intelligence, Drug-resistant epilepsy, Epilepsy surgery, Epileptognenesis, Focal cortical dysplasia, Hemimegaloencephaly, Mesial temporal sclerosis, Minimally invasive surgery, Neuroimaging, Neuroplasticity, Pediatric epilepsy, Pharmaco-resistance, Surgical outcomes

## Abstract

Meckel’s Pediatric drug-resistant epilepsy (DRE) is a significant neurological disorder that develops when seizures persist despite treatment with two appropriately selected antiseizure medications. Although only a subset of children with epilepsy develops DRE, prolonged uncontrolled seizures during childhood can result in irreversible cognitive, behavioral, and developmental impairment. Over time, growing evidence has demonstrated that epilepsy surgery is an effective treatment for appropriately selected patients and that earlier surgical intervention is associated with improved relief from seizures and long-term neurodevelopmental outcomes. This review examines the mechanisms underlying pharmaco-resistance, including network reorganization, neuroinflammation, blood-brain barrier dysfunction, and structural abnormalities that contribute to epileptogenesis. Common surgically remediable causes of pediatric DRE, including focal cortical dysplasia, mesial temporal sclerosis, tuberous sclerosis complex, hemimegaloencephaly, and tumor-associated epilepsy, are discussed along with current surgical approaches, including resective, disconnective, and minimally invasive procedures. The evidence comparing early versus delayed surgical intervention is reviewed with emphasis on seizure control, cognitive development, language, behavior, and quality of life. Finally, this review highlights persistent barriers to timely surgical referral, including socioeconomic, geographic, and racial disparities, while exploring emerging advances in neuroimaging, artificial intelligence-assisted lesion detection, and imaging biomarkers that may improve early diagnosis and patient selection. Collectively, the available evidence supports earlier referral for surgical evaluation in children with DRE and suggests that prompt intervention may preserve neurodevelopment, improve long-term functional outcomes, and maximize quality of life.

## 1. Introduction

Drug-resistant epilepsy (DRE), also referred to as refractory epilepsy, can arise from a variety of etiologies including infections, genetic abnormalities, and metabolic or structural defects [1]. It is defined by the failure to achieve seizure control despite appropriate treatment with antiseizure medications (ASMs) [2]. This lack of response is seen in up to 20–30% of children with epilepsy, making DRE a significant clinical concern in the pediatric population [1]. While pediatric epilepsy overall encompasses a wide range of causes, DRE is more commonly associated with structural, genetic, or developmental abnormalities such as focal cortical dysplasia, tuberous sclerosis complex, and perinatal brain injury [1].

At a mechanistic level, several factors contribute to pharmaco-resistance [3]. Alterations in ion channels or neurotransmitter receptors may reduce the effectiveness of ASMs, while over time, recurrent seizures can lead to maladaptive rewiring of neural circuitry [3]. This process promotes the formation of self-sustaining epileptic networks that are less responsive to pharmacologic intervention [4]. As a result, epilepsy evolves from a focal abnormality into a broader network disorder, further complicating treatment [4].

The consequences of prolonged, uncontrolled seizures in children can be severe, including cognitive impairment, developmental delay, and an increased risk of premature mortality, including sudden unexpected death in epilepsy (SUDEP). Although epilepsy surgery remains the only potentially curative treatment for DRE, other therapeutic options such as continued ASM therapy, the ketogenic diet, and vagus nerve stimulation may help reduce seizure burden [1]. However, these approaches are often insufficient in achieving complete seizure freedom.

Timely intervention is therefore critical in pediatric DRE. Current national and international guidelines recommend referral for surgical evaluation as soon as drug resistance is identified, defined as the failure of two appropriately chosen and tolerated ASM regimens to achieve seizure freedom [2]. Increasing advocacy for early surgical intervention is supported by evidence demonstrating improved seizure control and better neurodevelopmental outcomes when surgery is performed earlier in the disease course. Despite its high success rates, epilepsy surgery is often delayed due to concerns about potential complications, including neurological deficits, infection, hemorrhage, hydrocephalus, and the possibility of seizure recurrence [1].

## 2. Mechanisms of Drug Resistance and Surgical Rationale

### 2.1 Network reorganization

Epileptogenesis, the process by which a normal brain becomes epileptic, involves dysfunction of the brain’s neural networks at multiple levels [3]. The structural network of the brain refers to the anatomical connections between different neuronal populations, which allow for coordinated propagation of action potentials [4]. When this network is disrupted—whether from cortical malformations, hypoxic-ischemic injury, infection, or genetic mutations—it can lead to the development of a hyperexcitable region known as the epileptogenic focus [4].

After this initial injury, there is typically a latent phase in which overt seizures may not yet be present, but significant changes are occurring at the cellular and molecular level. During this period, the brain is essentially “rewiring” itself in a maladaptive way. Neuroinflammation plays a major role in this process, with activation of microglia and astrocytes and the release of pro-inflammatory cytokines such as TNF-α and interleukin-1β. These inflammatory mediators increase neuronal excitability and contribute to ongoing cellular stress and injury.

At the same time, disruption of the blood–brain barrier (BBB) begins to occur. Breakdown of tight junctions allows serum proteins and peripheral immune cells to enter the central nervous system, further amplifying inflammation [3,6]. This contributes to a cycle in which inflammation promotes excitability, and increased excitability leads to additional neuronal injury and further inflammatory signaling [3,6].

As epileptogenesis progresses, structural and functional network reorganization becomes more pronounced. Neurons begin to form abnormal connections through processes such as axonal sprouting, leading to recurrent excitatory circuits that can sustain seizure activity. In parallel, there is loss or dysfunction of inhibitory interneurons, resulting in a shift in the balance between excitation and inhibition toward a hyperexcitable state. Synaptic remodeling also occurs, with increased glutamatergic signaling and impaired GABAergic inhibition, further lowering the seizure threshold.

Over time, these changes allow epileptogenic activity to extend beyond the original focus, resulting in the formation of distributed seizure networks [4]. This transition from a focal abnormality to a network-level disorder is a key factor in the development of drug-resistant epilepsy. At this stage, antiseizure medications are often less effective, as they target individual neuronal mechanisms rather than the broader, reorganized network driving seizure propagation [4].

### 2.2 Structural lesions

Structural abnormalities of the brain are one of the most common underlying causes of drug-resistant epilepsy in the pediatric population [1]. These lesions create areas of abnormal cortical organization that cause neurons to become prone to hyperexcitability and recurrent seizure activity [6]. In contrast to more generalized epilepsies, structural lesions often produce focal seizures that originate from a specific region of the brain, making them especially relevant in cases of drug resistance and surgical intervention [1].

Some of the most identified structural causes of pediatric DRE include focal cortical dysplasia, tuberous sclerosis complex, low-grade epilepsy-associated tumors, and injuries related to hypoxic-ischemic events or perinatal stroke [1]. Among these, focal cortical dysplasia is one of the most frequent in children undergoing epilepsy surgery. These lesions are characterized by disrupted cortical layering and abnormal neuronal morphology, which impair normal electrical signaling and promote synchronous, hyperexcitable firing patterns [7].

At a cellular level, structural lesions contribute to seizure generation through a variety of mechanisms. Abnormal neurons within these regions often exhibit altered ion channel function and increased excitatory neurotransmission [6]. In addition, there is frequently a reduction in inhibitory interneuron activity, resulting in the promotion of excitation [6]. These changes create a localized environment that is highly susceptible to generating and sustaining epileptic discharges.

Structural lesions also play a significant role in the development of network reorganization [4]. Recurrent seizure activity in these abnormal regions promotes synaptic remodeling and the formation of atypical neural connections, allowing epileptogenic activity to spread beyond the initial lesion [4]. Over time, this leads to the transition from a focal epileptogenic zone to a more distributed epileptic network, which is more difficult to control with medication alone [4].

From a clinical standpoint, the presence of a well-defined structural lesion is one of the strongest predictors of drug resistance, but it is also associated with better surgical outcomes when appropriately localized and resected [1]. Advances in neuroimaging, particularly high-resolution magnetic resonance imaging (MRI), have improved the ability to detect subtle lesions and guide surgical planning [9]. As a result, early identification of structural abnormalities is crucial in determining which patients may benefit from surgical intervention and improving long-term outcomes in pediatric DRE [5].

### 2.3 Neuroinflammation

Neuroinflammation is a key contributor to drug-resistant epilepsy (DRE), with microglia and astrocytes playing central roles in this process [6–8]. These glial cells become activated in response to neuronal injury and release a variety of cytokines that regulate and amplify inflammatory signaling within the brain [6,8–13]. Studies have demonstrated elevated levels of pro-inflammatory mediators, such as tumor necrosis factor-alpha (TNF-α), and activation of nuclear factor kappa B (NF-κB), a major inflammatory signaling pathway [3,14–19]. The presence of these markers suggests that brain tissue in DRE exists in a state of chronic inflammation [3].

This inflammatory activity is most prominent within the epileptogenic zone, the region of the brain responsible for generating seizures due to its altered excitability [6]. However, it is important to recognize that the epileptogenic zone is part of a broader network of functionally defined regions, including the seizure onset zone, irritative zone, symptomatogenic zone, functional deficit zone, and the structural lesion or epileptogenic damage zone [6]. Accurate localization of these regions, often through electroencephalography (EEG) and advanced imaging techniques, is essential in determining candidacy for surgical intervention [1,20].

In addition to elevated pro-inflammatory cytokines, alterations in other immune mediators such as interleukin-2 (IL-2) have also been observed [3,6]. IL-2 primarily functions in T-cell activation and regulation of the adaptive immune response, and dysregulation of this pathway may contribute to sustained neuroinflammatory signaling in DRE [3,6,18]. Through its role in immune activation, IL-2 can indirectly promote the release of additional cytokines, including TNF-α and interleukin-1β, further amplifying inflammation within the central nervous system [3,6,14–19].

A major downstream consequence of this inflammatory cascade is disruption of the blood–brain barrier (BBB) [3]. Inflammatory mediators impair endothelial tight junction proteins such as claudins and occludin, leading to increased BBB permeability [3]. This allows peripheral immune cells and serum proteins to enter the brain, further exacerbating local inflammation [3].

BBB dysfunction also contributes to changes in neuronal excitability, partly through astrocyte activation and increased glutamatergic signaling, ultimately lowering the seizure threshold [3,6,18].

Additionally, inflammation-induced BBB alterations have important implications for pharmaco-resistance. Upregulation of efflux transporters at the BBB, such as P-glycoprotein, can reduce the effective concentration of antiseizure medications within epileptogenic tissue, even when systemic drug levels are adequate [3]. Together, these findings highlight how neuroinflammation, through cytokine signaling, BBB disruption, and immune activation, contributes to both seizure propagation and the development of drug-resistant epilepsy [3,6,14–18].

## 3. Rationale for Surgery

### 3.1 Disconnection of seizure networks

While seizures might originate from a particular epileptogenic zone, they are dependent upon the ability of the action potential to propagate through interconnected pathways in the brain [4]. Because of this, modern surgical approaches have altered from mainly targeting lesion removal to also focusing on the disruption of the entire seizure network [4].

Seizure propagation relies on functional and structural connectivity between regions of the brain, as these connections allow epileptic activity to spread beyond the initial focus and recruit additional regions [4]. In cases of DRE in children, these networks become well established over time due to ongoing seizure activity and maladaptive neuroplasticity [4]. Disconnection strategies aim to interrupt these pathways rather than remove all epileptogenic tissue [1]. The isolation of the seizure focus area from the rest of the brain prevented the synchronization and spread of epileptic activity [4]. This approach is especially useful in cases where the epileptogenic zone overlaps with cortices that, if injured or completely resected, would cause critical neurological deficit [1]. These regions are called eloquent cortex.

Several surgical techniques have been developed to achieve network disconnection [1]. For example, corpus callosotomy is an approach that can be performed with radiosurgery and involves the disconnection of the epileptic network between the two cerebral hemispheres [21]. This approach is primarily reserved for patients who have medically intractable epilepsy with multifocal regions of epileptic activity and are not candidates for focal resection [21]. Another disconnection procedure is called functional hemispherectomy, which is utilized in cases in which the seizure foci diffusely localize to one hemisphere [22]. Functional hemispherectomy aims to prevent the propagation of seizures to the contralateral hemisphere [22]. Finally, multiple subpial transection (MST) targets intracortical propagation pathways, and is especially useful in cases where eloquent cortex cannot be resected [23]. This technique utilizes small, shallow cuts under the pia mater, interrupting horizontal cortical fibers while leaving the vertical input/output pathways intact [23]. As a result, the neurons can still function normally, but they cannot synchronize into a seizure. This allows for the preservation of motor function and language. However, MST is not always curative and is considered a palliative epilepsy approach, meaning that it often reduces seizure frequency but does not eliminate it [23].

### 3.2 Plasticity in pediatric brain

Neuroplasticity plays a key role in the pediatric brain’s ability to adapt to both the presence of epilepsy and the process of surgical intervention [24,25–28]. In children with DRE, the process of reorganization of functional brain networks is an adaptive response in which the brain is attempting to preserve critical functions in the presence of pathological disruption [24,27,28].

An example of this phenomenon is the reorganization of language networks [24]. Typically, language is lateralized to the left hemisphere of the brain [24]. However, in pediatric patients with focal epilepsy, particularly when the epileptogenic zone involves eloquent regions, the lateralization can be altered. Previous case studies have shown that the language function can reorganize towards homologous regions on the contralateral hemisphere as a compensatory mechanism [24]. This shift is not solely functional but also structural [24].

## 4. Common Surgical Pathologies in Pediatric Epilepsy

### 4.1 Focal cortical dysplasia

Focal cortical dysplasia (FCD) is a malformation of cortical development characterized by disrupted neuronal migration during embryogenesis, resulting in a disorganized, structurally abnormal cortical architecture [29]. Neurons fail to migrate and differentiate appropriately, leading to dysmorphic cells, aberrant cortical layering, and poorly organized synaptic connectivity [29]. These abnormalities contribute to intrinsic hyperexcitability, an imbalance between excitatory and inhibitory signaling, and dysfunctional cortical circuitry, ultimately promoting the formation of epileptogenic networks [7,29].

FCD is a major cause of Drug-resistant epilepsy in pediatric populations [29]. As a fixed structural lesion, it is not amenable to pharmacologic reversal [7]. While antiseizure medications (ASMs) can modulate neuronal excitability, they do not correct the underlying cortical malformation, allowing the epileptogenic focus to persist [7].

FCD is broadly classified into two main subtypes [29]. Type I is characterized by isolated cortical dyslamination with relatively mild architectural disruption [29]. It is often more subtle on neuroimaging and may be difficult to detect, though it can still be epileptogenic [29]. In contrast, Type II FCD is the classic and more severe form, characterized by dysmorphic neurons with or without balloon cells. It is highly epileptogenic and represents one of the leading indications for pediatric epilepsy surgery [29].

On MRI, Type II FCD commonly demonstrates cortical thickening, blurring of the gray-white matter junction, and the characteristic transmantle sign [20]. The transmantle sign appears on T2-weighted or FLAIR imaging as a tapering band of hyperintensity extending from the lateral ventricle through the subcortical white matter to the cortical surface [20]. This finding reflects abnormal radial neuronal migration and the presence of disorganized cells and gliosis along this pathway [20,29]. Identification of the transmantle sign is clinically significant, as it improves localization of the epileptogenic zone and is associated with more favorable surgical outcomes due to the presence of a well-defined anatomical target [20].

### 4.2 Mesial Temporal Sclerosis

Mesial Temporal Sclerosis (MTS) is an abnormality in the structure of the mesial temporal lobe, with the hippocampus being the main structure affected [30]. It is difficult to pinpoint the exact cause of MTS, but it is believed to be caused by things such as central nervous system infections, traumatic brain injury, and congenital malformation [30]. MTS causes seizures via reactive scarring of the brain and, like many other seizure pathologies, reorganization of the synaptic circuits in the mesial temporal region [30].

### 4.3 Tuberous Sclerosis

Tuberous sclerosis is a genetic disorder that creates multiple epileptogenic regions throughout the brain [31]. Tuberous sclerosis complex (TSC) is an autosomal dominant neurocutaneous disorder that can cause benign tumors to form in a variety of organ systems, including the heart, kidneys, eyes, lungs, skin, and brain [31]. TSC is caused by mutations in either the *TSC1* or *TSC2* genes, which encode the proteins hamartin and tuberin, respectively. These proteins are responsible for regulating the mTOR pathway, which controls cell growth and proliferation, protein synthesis, and neuronal development [31]. The primary epileptogenic lesions associated with TSC are known as cortical tubers, regions of disorganized cortical architecture, abnormal neuronal connectivity, and gliosis that are functionally similar to focal cortical dysplasia [31]. Several factors contribute to the development of drug-resistant epilepsy in patients with TSC, including the presence of multiple seizure foci, early seizure onset, persistent structural abnormalities, and hyperactivation of the mTOR pathway [31]. Unlike focal cortical dysplasia, which typically involves a single lesion, TSC often produces numerous cortical tubers capable of generating seizures and forming widespread epileptogenic networks [31]. However, targeted therapies have emerged as a treatment option. Everolimus, an mTOR inhibitor, reduces mTOR signaling and has been shown to decrease seizure frequency in some patients with TSC-associated epilepsy [32].

### 4.4 Hemimegaloencephaly

Hemimegaloencephaly (HME) is a congenital malformation that causes severe overgrowth of one cerebral hemisphere [33]. HME is believed to be caused by somatic mutations affecting the mTOR pathway, the same mechanism that is affected in TSC [33]. Similarly, this mutation leads to excessive neuronal growth, disorganized cortical structure, and abnormal cell proliferation, all of which lead to epilepsy [33]. These physical structural changes to one hemisphere are the reason why HME is resistant to drugs and ASMs, and as a result, HME is a classical indication for hemispherectomy or hemispherectomy surgical procedure [33]. This involves the disconnection of the abnormal hemisphere from the unaffected one, allowing the discontinuation of the seizure network to reduce seizure burden [22]. Although it is considered an aggressive treatment option, the neuroplasticity of pediatric brains allows for the unaffected hemisphere to compensate for the many of the functions of the affected hemisphere over time [24]. Time is of the essence when it comes to this surgical option, so the earlier the surgery is performed, the better the long-term outcome [5].

### 4.5 Tumor-associated epilepsy

Tumor-associated epilepsy represents another important structural cause of pediatric drug-resistant epilepsy [34]. In contrast to developmental malformations such as focal cortical dysplasia or hemimegaloencephaly, tumor-associated epilepsy arises from neoplastic lesions that disrupt normal cortical architecture and promote neuronal hyperexcitability [34]. The tumors most implicated in pediatric epilepsy are low-grade cortical neoplasms, including gangliogliomas, neuroepithelial tumors, and low-grade gliomas [34]. Seizure generation occurs through multiple mechanisms, including cortical irritation, increased excitatory signaling, reduced inhibitory activity, chronic inflammation, and the development of epileptogenic networks within the surrounding cortex [34]. Because antiseizure medications do not eliminate the underlying lesion, tumor-associated epilepsy may become drug resistant. However, surgical resection often provides excellent seizure outcomes, particularly when both the tumor and adjacent epileptogenic cortex can be completely removed [35].

**Figure 1:**
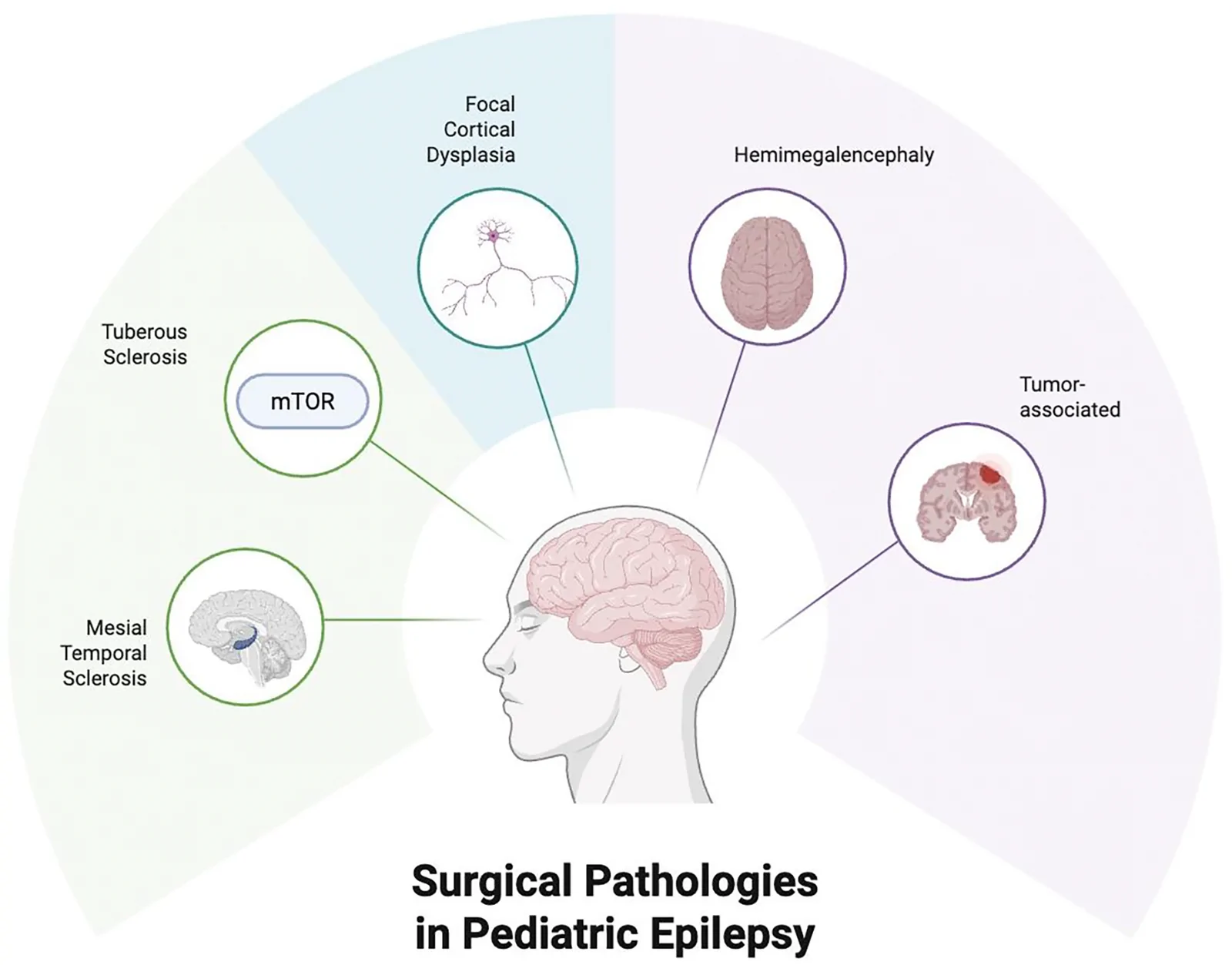
A brief overview of the surgical pathologies in pediatric epilepsy. Created with BioRender.com

There are a variety of pathologies that cause pediatric epilepsy and indicate a need for surgical remedy. Mesial Temporal Sclerosis is one that involves a structural abnormality in the hippocampus (Figure 1) [30]. Tuberous Sclerosis is another pathology that is characterized as an autosomal dominant neurocutaneous disorder that can cause benign tumors to form in a variety of organ systems [31]. Tuberous Sclerosis becomes epileptogenic primarily through its impact on the mTOR pathway, which controls neuronal development [31]. Focal Cortical Dysplasia is a result of neurons in the brain failing to migrate and differentiate properly during the developmental stage of embryogenesis [28]. Hemimegaloencephaly is caused by the overgrowth of one cerebral hemisphere, resulting in a lack of cortical organization and development of epileptogenic networks [33]. Tumor-associated epilepsy arises from neoplastic lesions on the brain that promote neuronal hyperexcitability [34,35]. All these pathologies are structural issues within the brain; therefore, antiseizure medications are not effective solutions, and surgery may be necessary to treat these cases [1].

## 5. Types of Surgical Interventions

### 5.1 Resective

Resective surgeries for pediatric DRE are focused on removing the seizure-producing tissue [1]. These surgeries are safest when the epileptogenic zone can be clearly localized and removed to reduce the likelihood of neurological deficits [1]. Cases in which resective surgery options are indicated are focal cortical dysplasia, mesial temporal sclerosis, tumor-associated epilepsy, and certain cases of tuberous sclerosis complex [1]. Examples of the procedures include lesionectomy, focal cortical resection, lobectomy, and temporal lobectomy [1]. Resection offers the greatest chance of complete seizure freedom when the epileptogenic is completely removed, but this form of treatment may not be effective if the seizures originate from an eloquent cortex or when the network is dispersed and multifocal [1,4].

### 5.2 Disconnective

Disconnective surgeries are used to leave the epileptogenic tissues of the brain in place but isolate it from the other regions [4]. This type of procedure is reserved for the patient population in which complete resection of the epileptogenic tissue is not possible due to the affected region being too large, overlapping with eloquent cortex, or spanning an entire hemisphere [1]. One common disconnective procedure is called the corpus callosotomy, which is performed by sectioning part of the corpus callosum to prevent seizures from spreading between hemispheres [21]. Another common procedure is the hemispherotomy/hemispherectomy, which involves the disconnection of the malformed hemisphere from the healthy one [22]. This type of surgery is used for cases of seizures caused by hemimegaloencephaly, encephalitis, and large hemispheric malformations [22].

### 5.3 Minimally invasive

Minimally invasive surgery aims to achieve the same goals as disconnective and resective surgery but reduces the surgical disruption to the epileptogenic zone [1]. The innovation of minimally invasive procedures has come with advances in imaging and radiology techniques allowing for treatment of select patients [20]. Laser interstitial thermal therapy (LITT) is performed by inserting a laser probe to do an MRI-guided thermal ablation of epileptogenic tissue [36]. This type of procedure is commonly used in patients with MST, hypothalamic hamartomas, and small focal lesions [36]. The advantages of LITT are a shorter hospital stay, shorter postoperative recovery time, and a smaller incision site [36]. Radiosurgery is another option of minimally invasive surgery used to treat DRE and uses focused radiation without open surgery [21]. This option is reserved for deep epileptogenic lesions on the brain but is not commonly used due to the reduction of seizures taking months to occur [21].

**Figure 2:**
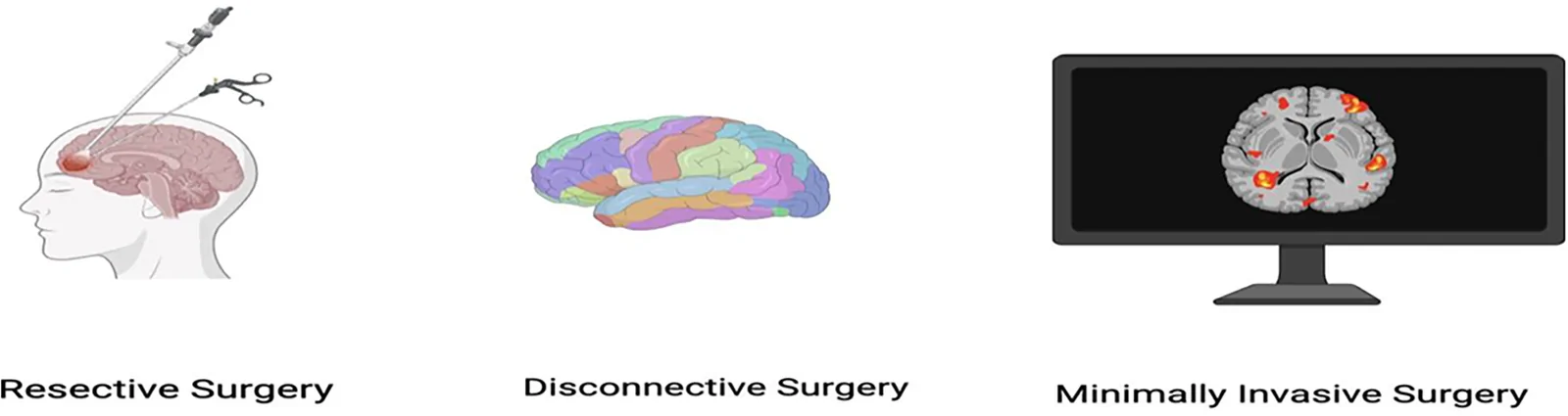
Examples of the options for surgery in pediatric epilepsy. Created with BioRender.com

Figure 2 presents some of the options for surgery regarding pediatric epilepsy. Resective surgery is an aggressive option that involves removing the physical tissue in the region of the brain containing the lesion [1]. This route offers the greatest chance of seizure freedom for patients [37]. Disconnective surgery is the procedure of choice for patients whose lesions show complications that contraindicate the resective surgery option [1]. These procedures leave the tissues of the brain in place but disconnect isolate one of the regions colored in the figure from the others to prevent propagation of the epileptic network [4,21,22]. Finally, minimally invasive surgery involves using imaging such as MRI to guide a laser probe to perform an ablation of epileptogenic tissue [36].

## 6. Outcomes of Early vs. Delayed Surgery

There is increasing evidence that the decision to implement earlier surgical intervention in appropriately selected pediatric patients is correlated with improved seizure freedom, developmental outcomes, cognitive trajectory, and quality of life [37]. In the past, surgery was considered a last resort form of treatment after years of failed medical therapy, but the growing recognition and understanding of the detrimental effects of persistent seizures on the developing brain has shifted the standard of medical practice towards earlier surgical intervention [5].

### 6.1 Seizure freedom rates

The most consistently reported benefit of early surgery is an increased likelihood of seizure freedom [37]. Children with well localized epileptogenic lesions such as focal cortical dysplasia, mesial temporal sclerosis, tumor associated epilepsy, or hemispheric malformations have the highest chance of achieving favorable seizure outcomes following surgery [37]. Earlier intervention may prevent the expansion of epileptogenic networks that can occur with years of uncontrolled seizures [4]. As recurrent seizures recruit neighboring cortical regions into the epileptogenic network, successful seizure control may become increasingly difficult to achieve [4]. Several studies have demonstrated that shorter epilepsy duration prior to surgery is associated with improved postoperative seizure outcomes, suggesting that intervention before extensive network reorganization occurs may provide the greatest benefit [37].

### 6.2 Developmental outcomes

Childhood represents a period of rapid neurological development characterized by ongoing synaptogenesis, circuit refinement, and acquisition of cognitive and motor skills [24].

Frequent seizures during this period can interfere with normal developmental processes [37]. Children who undergo surgery earlier in the course of their disease often demonstrate improved developmental progress compared with those who experience prolonged periods of uncontrolled epilepsy [38]. This benefit is particularly evident in infants and young children with persistent seizures that may contribute to developmental stagnation or regression [38]. Importantly, successful seizure control may allow children to resume developmental trajectories that were previously disrupted by ongoing epileptic activity [38].

### 6.3 Cognitive trajectory

Chronic epilepsy during childhood can negatively affect cognitive development, language acquisition, academic performance, and behavioral regulation [38]. Recurrent seizures, medication burden, and underlying structural abnormalities may all contribute to deficits in attention, memory, executive function, and social development [38]. While surgery cannot reverse all existing impairments, early intervention may prevent further decline and improve long-term developmental outcomes [38]. Language development is particularly vulnerable during childhood, but the pediatric brain possesses a remarkable capacity for neuroplasticity [24]. Following surgery, language functions may reorganize to other regions of the brain, especially in younger children [24]. This ability decreases with age, providing an additional advantage to earlier intervention [24]. Successful seizure control has also been associated with improvements in behavior, emotional regulation, social interactions, and school performance [39]. Overall, earlier surgery may help preserve cognitive function, support language development, and improve behavioral outcomes by limiting the cumulative effects of uncontrolled seizures on the developing brain [38,39].

### 6.4 Quality of Life

Quality of life represents perhaps the most comprehensive measure of surgical success [40]. Children who achieve seizure freedom frequently experience improvements in independence, educational attainment, social participation, physical safety, and emotional well-being [40]. Caregivers also report reduced stress, improved family functioning, and decreased caregiving burden following successful surgical intervention [40]. Quality of life improvements may occur even when complete seizure freedom is not achieved [41]. Significant reductions in seizure frequency and severity can still provide meaningful benefits for both patients and families [41].

**Figure 3:**
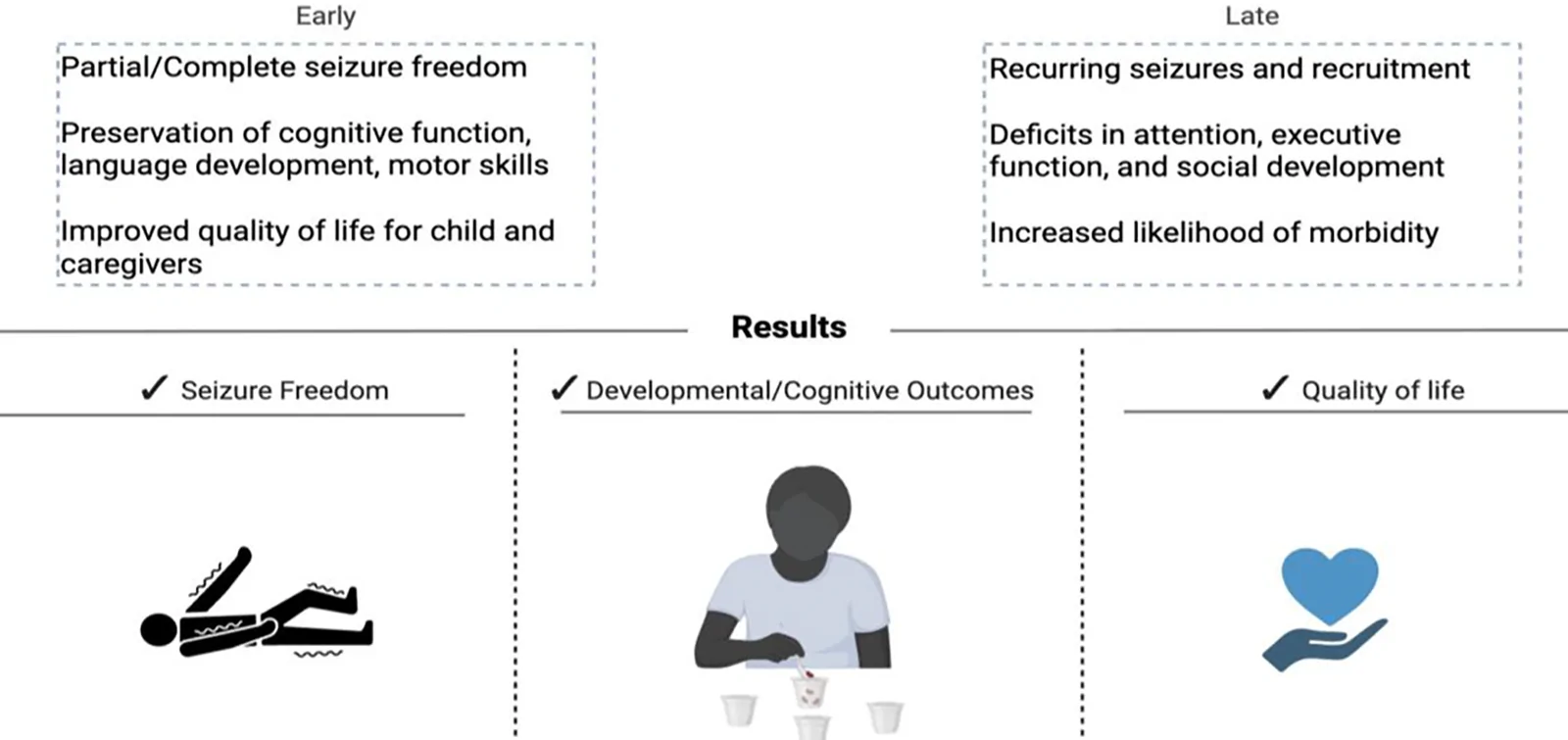
Schematic diagram showing the early and late timing outcomes following surgery in pediatric epilepsy.

A crucial factor in the outcomes of surgery for pediatric epilepsy is the timing of the referral/procedure (Figure 3). Studies have found that the earlier the process gets started when surgery is indicated as the form of treatment, the better the outcome [2,5]. Some of these positive outcomes can be either a partial or complete relief from seizures, improved outcomes of cognitive development and motor skills, and better overall quality of life for both the child and the caregivers [37,38,40]. On the other hand, the negative outcomes caused by delayed referral for surgery can be persistent seizures and ongoing recruitment of epileptogenic networks, impaired cognitive development, and increased likelihood of death [4,37].

### 6.5 Risks and complications

Although epilepsy surgery can provide significant benefits for children with drug-resistant epilepsy, it is not without risk [1]. Potential complications include surgical morbidity, neurological deficits, infection, and psychosocial challenges [42]. The type and severity of these risks vary depending on the procedure performed, the location of the epileptogenic zone, and the patienťs underlying condition [42].

### 6.6 Surgical morbidity

As with any neurosurgical procedure, epilepsy surgery carries risks associated with anesthesia and operative intervention [42]. Potential complications include hemorrhage, cerebrospinal fluid leaks, wound complications, and the need for additional procedures [42]. Major complications are relatively uncommon, but they remain an important consideration during surgical planning [42].

### 6.7 Neurological deficits

The most significant concern following epilepsy surgery is the possibility of new neurological deficits [42]. Depending on the location of the resection or disconnection, patients may experience impairments in motor function, sensation, vision, language, or memory [26]. Advances in neuroimaging, functional mapping, and intraoperative monitoring have helped reduce these risks, but they cannot be eliminated entirely [20,42].

### 6.8 Infection

Postoperative infections, including wound infections and meningitis, represent another potential complication [42]. Although infection rates are generally low, these complications may prolong hospitalization and require additional medical treatment [42].

### 6.9 Psychosocial impact

Epilepsy surgery can also have psychosocial consequences for both patients and families [43]. Recovery from surgery, changes in daily routines, and anxiety surrounding outcomes may create emotional stress [43]. Additionally, some children may require ongoing rehabilitation or educational support following surgery [43]. Despite these challenges, many patients and families ultimately report improved quality of life following successful seizure control [40,43].

### 6.10 Timing Controversy/Barriers to Surgical Referral

Despite the growing evidence suggesting that earlier surgical referral times correlates with better surgical outcomes with pediatric DRE, many children continue to experience major delays in referral for surgical evaluation [2,37]. Some of these include but are not limited to the timing of referral, age considerations and neuroplasticity, socioeconomic barriers, geographic access, and racial disparity [2].

### 6.11 When to refer?

There are still some lingering concerns about surgery in very young patients when it comes to DRE, and rightfully so [2]. Many of these procedures require long and dangerous resections that have the potential to cause more harm than good [42]. This causes delay and debate as to when is the best time to refer for surgical consultation, with some physicians choosing to play it safe rather than put a child at risk of surgical morbidity [2].

### 6.12 Socioeconomic barriers/insurance issues

Access to epilepsy surgery may be limited by socioeconomic constraints, insurance coverage issues, and difficulty obtaining referrals to specialized epilepsy centers [44]. These barriers can have a negative impact on the timing of evaluation and treatment, even when surgery may be clearly clinically indicated [44].

### 6.13 Geographic access

Specialized pediatric epilepsy centers often are found within large academic medical centers, requiring families to travel long distances and uproot their lives to receive evaluation and treatment [44]. These geographic limitations may contribute to delayed referrals and reduced access to surgical care.

### 6.14 Racial disparity

Studies have demonstrated disparities in access to epilepsy surgery among racial and ethnic minority populations [45]. Children from underserved communities are often less likely to receive timely referral, comprehensive evaluation, or surgical treatment despite similar clinical indications [45]. These disparities highlight the need for improved access and equity in epilepsy care.

## 7. Future Directions and Conclusion

### 7.1 Early imaging biomarkers

One of the greatest challenges remains identifying which children are most at risk for developing pediatric DRE before years of unsuccessful treatment have passed [2,5]. Advances in neuroimaging may allow physicians to detect DRE abnormalities earlier and with greater precision than in the past [20].

Conventional MRI remains the cornerstone of presurgical evaluation, but many lesions can be subtle and nearly invisible on routine MRI imaging [20]. Emerging variations of MRI such as high-resolution structural imaging, diffusion tensor imaging, functional MRI, and connectome analysis are improving the detection of DRE abnormalities [20]. There is an increasing amount of investigational research into the imaging biomarkers capable of locating network-level dysfunction before it becomes clinically apparent [20]. These biomarkers may assist with earlier detection of children at high risk for developing DRE and facilitate earlier intervention.

Beyond lesion detection, future imaging approaches may provide insight into how epileptogenic networks evolve over time [20]. Understanding the progression of these networks could help clinicians determine which patients are likely to benefit from earlier surgical treatment and which may respond to alternative therapies [4,20].

### 7.2 Lesion detection with Artificial Intelligence

Artificial intelligence (AI) is playing an increasing role as powerful tools for epilepsy diagnosis and surgical planning [46]. Many structural causes of DRE, such as focal cortical dysplasia, can be difficult for even the most experienced neuroradiologists to detect [46]. AI-based image analysis systems have demonstrated success in finding subtle cortical abnormalities that may otherwise go unrecognized.

Artificial intelligence and machine learning platforms can analyze large imaging datasets and identify patterns that are often imperceptible to the human eye [47]. These technologies have the potential to improve diagnostic accuracy, reduce delays in surgical evaluation, and increase identification of surgically treatable lesions [46–48]. As AI systems continue to improve, they may become valuable adjuncts in the presurgical evaluation process, particularly for diagnostically challenging cases [48–50].

## 8. Summary

Pediatric drug-resistant epilepsy represents a significant neurological disorder that can profoundly affect cognitive development, language acquisition, behavior, academic performance, and overall quality of life [37,38,40]. Structural abnormalities such as focal cortical dysplasia, mesial temporal sclerosis, tuberous sclerosis complex, hemimegaloencephaly, and tumor-associated epilepsy are among the most common causes of medically refractory seizures in children [1]. Advances in neuroimaging, surgical techniques, and our understanding of epileptogenic networks have dramatically expanded treatment options for these patients [1,4,20].

A growing body of evidence supports the role of surgical intervention as an effective treatment for appropriately selected children with drug-resistant epilepsy [37]. Resective, disconnective, and minimally invasive procedures can provide substantial reductions in seizure burden and, in many cases, achieve long-term seizure freedom [37]. Importantly, earlier intervention has been associated with improved developmental, cognitive, behavioral, and quality-of-life outcomes, likely reflecting the prevention of ongoing neurological injury during critical periods of brain maturation [38–40].

As imaging technology, artificial intelligence, and network-based approaches continue to evolve, the ability to identify and treat surgically remediable epilepsy is likely to improve further [4,20,46,47]. While challenges related to access, referral patterns, and healthcare disparities remain, increasing recognition of the benefits of early evaluation may help reduce delays in treatment [2,44,45]. Ultimately, the management of pediatric drug-resistant epilepsy is shifting from a model focused solely on seizure control toward one centered on preserving neurodevelopment, maximizing quality of life, and improving lifelong outcomes for affected children [1,37,38,40].

## 9. Key Points

The key findings, outstanding questions, and challenges identified in this review are:

- Early surgical intervention in pediatric drug-resistant epilepsy is associated with higher rates of seizure freedom and improved long-term neurodevelopmental outcomes compared with delayed surgery.
- Structural lesions, particularly focal cortical dysplasia, mesial temporal sclerosis, tuberous sclerosis complex, hemimegaloencephaly, and low-grade epilepsy-associated tumors, are among the most common causes of pediatric drug-resistant epilepsy.
- Drug resistance develops through multiple mechanisms, including network reorganization, neuroinflammation, blood-brain barrier dysfunction, and irreversible structural abnormalities that cannot be corrected with antiseizure medications alone.
- Advances in neuroimaging have improved lesion detection and surgical planning, allowing more children with previously “MRI-negative” epilepsy to become surgical candidates.
- Resective, disconnective, and minimally invasive surgical techniques can all provide excellent seizure control when appropriately matched to the patienťs epileptogenic focus and functional anatomy.
- Earlier surgery takes advantage of the developing brain's neuroplasticity, improving cognitive, language, behavioral, and overall developmental outcomes while limiting the cumulative effects of ongoing seizures.
- Although epilepsy surgery carries risks including neurological deficits, infection, hemorrhage, and psychosocial challenges, complication rates remain relatively low in experienced pediatric epilepsy centers.
- Many children continue to experience delayed referral for epilepsy surgery because of socioeconomic barriers, geographic limitations, insurance restrictions, and persistent racial disparities in access to specialized care.
- Can emerging technologies such as advanced MRI techniques, functional imaging, and artificial intelligence improve the early detection of subtle epileptogenic lesions and increase the number of children eligible for curative surgery?
- How can healthcare systems reduce disparities in referral patterns and ensure that all children with drug-resistant epilepsy receive timely evaluation at comprehensive pediatric epilepsy centers?
